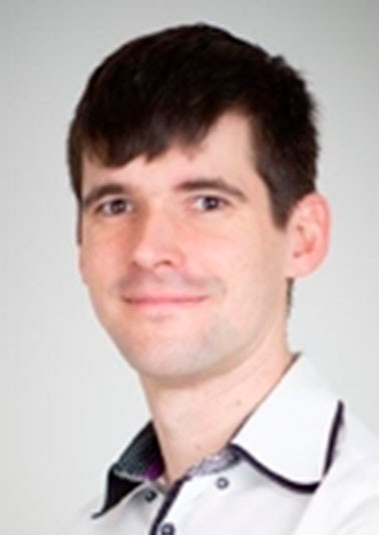# Cheminformatics in Natural Product‐based Drug Discovery

**DOI:** 10.1002/minf.202000171

**Published:** 2020-09-06

**Authors:** Ya Chen, Johannes Kirchmair

**Affiliations:** ^1^ Center for Bioinformatics (ZBH) Department of Computer Science Faculty of Mathematics Informatics and Natural Sciences Universität Hamburg 20146 Hamburg Germany; ^2^ Department of Pharmaceutical Chemistry Faculty of Life Sciences University of Vienna 1090 Vienna Austria

**Keywords:** cheminformatics, natural products, drug discovery, databases, in silico methods

## Abstract

This review seeks to provide a timely survey of the scope and limitations of cheminformatics methods in natural product‐based drug discovery. Following an overview of data resources of chemical, biological and structural information on natural products, we discuss, among other aspects, in silico methods for (i) data curation and natural products dereplication, (ii) analysis, visualization, navigation and comparison of the chemical space, (iii) quantification of natural product‐likeness, (iv) prediction of the bioactivities (virtual screening, target prediction), ADME and safety profiles (toxicity) of natural products, (v) natural products‐inspired de novo design and (vi) prediction of natural products prone to cause interference with biological assays. Among the many methods discussed are rule‐based, similarity‐based, shape‐based, pharmacophore‐based and network‐based approaches, docking and machine learning methods.

## Graphical abstract

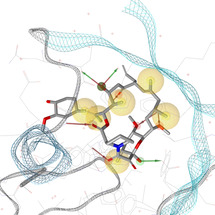

## 1 Introduction

Natural products (NPs) have a long record of use as components of traditional medicines and herbal remedies. Even for modern small‐molecule drug discovery they remain the single most prolific source of inspiration.^[1]^ In fact, about two‐thirds of all small‐molecule drugs approved between 1981 and 2019 are related, to different extents, to NPs.^[1]^ Whereas only 5 % of the drugs that have been introduced to the market during this timeframe are unaltered NPs, 28 % are NP derivatives, and 35 % mimic and/or contain a NP pharmacophore.^[1]^ A highly visible recognition of the relevance of NP‐research for public health is the award of the 2015 Nobel Prize in Physiology or Medicine to William C. Campbell, Satoshi Omura, and Youyou Tu for the discovery of two NPs (avermectin and artemisinin) that led to fundamental improvements in the treatment of diseases caused by parasites.

As a result of evolutionary processes, NPs have a wide range of bioactivities in different organisms. For this reason a substantial number of NPs are recognized as privileged structures.[^2^, ^3^] NPs are highly diverse in their molecular structures and physicochemical properties. Many of them have favorable ADME and physicochemical properties; others are clearly beyond what is generally considered as the drug‐like chemical space.[^4^, ^5^, ^6^] NPs can be highly complex in terms of molecular structure, in particular with regard to their 3D molecular shape, stereochemistry, ring complexity (macrocycles; bridged or fused ring systems) and conformational space (high number of rotatable bonds; low degree of aromaticity).[^7^, ^8^, ^9^] This poses fundamental challenges to 3D cheminformatics methods for which reasons the development of force fields and algorithms for the prediction of the protein‐bound conformations of such complex molecules remains one of the most actively pursued research topics in cheminformatics.[^10^, ^11^, ^12^, ^13^, ^14^, ^15^]

The real bottleneck of NP‐based drug discovery, however, is the availability of materials for testing. The sourcing process can be complex, lengthy and costly, and transport across borders may prove legally challenging.^[16]^ Once the material has arrived at its destination, the production of extracts, the in vitro testing for bioactivity, the identification and isolation of the bioactive compounds from these complex mixtures, the determination of the mode of action, the resupply of compounds of interest (e. g. through partial or total chemical synthesis), and the profiling of their pharmacological, pharmacokinetic and toxicological properties all require expertise, substantial efforts, time and funds, and there is no guarantee of success.[^4^, ^16^, ^17^]

Computational methods can make substantial contributions to NP‐based drug discovery and support experimentalists throughout the hit discovery, hit‐to‐lead and lead optimization phases.[^18^, ^19^] They have been shown to be particularly powerful, not just in identifying bioactive NPs, but also in prioritizing (plant) materials for testing,[^20^, ^21^, ^22^, ^23^] hence helping experimentalists to focus their resources on the most promising materials. Computational methods are also employed, for example, in (i) data curation and NP dereplication, (ii) chemical space analysis, visualization, navigation and comparison, (iii) quantification of natural product‐likeness, (iv) prediction of bioactivity spectra, ADME and safety profiles (toxicity), (iv) natural products‐inspired de novo design and (v) prediction of natural products prone to cause interference with biological assays.

Compared to the costs involved in experimental approaches, the funds required for in silico experiments seem almost negligible. An in‐house high‐performance computing facility is no longer essential. Today, calculations can be run (if at all needed) at very large scales in the cloud, at moderate cost and low complexity. Merely software license fees remain a substantial cost factor and have constantly increased throughout recent years. At the same time, we are now seeing a growing number of powerful open‐source tools becoming available, much like what has been quite common to the field of bioinformatics. Some of the most outstanding software in this context are RDKit^[24]^ and CDK[^25^, ^26^] (both are open‐source toolkits for cheminformatics), KNIME^[27]^ (an open‐source analytics platform), and scikit‐learn[^28^, ^29^] (an open‐source Python module for machine learning).

With this review, we aim to provide a succinct but comprehensive overview of the scope and limitations of cheminformatics methods in NP‐based drug discovery in a format that is accessible to researchers from different domains with an interest in drug discovery. The discussion covers a large number of state‐of‐the‐art methods in cheminformatics as well as data resources relevant to NP‐based drug discovery.

## 2 Natural Products Collections Relevant to Computer‐guided Natural Products Research

### 2.1 Virtual Natural Products Collections

The last decade has seen a steep increase in databases providing access to chemical, biological, pharmacological, toxicological and structural data on NPs. We recently conducted comprehensive surveys of databases that are particularly relevant to NP‐based drug discovery.[^6^, ^30^, ^31^] As a minimum requirement, any of the more than 30 databases surveyed feature a chemistry‐aware web interface for searching and browsing molecular structures. Most of the databases also offer free bulk download, enabling virtual screening and other applications. From these studies we gathered that the total number of NPs for which their structures can be obtained via bulk download from free databases is in excess of 250k, approaching 300k.

Unfortunately, the half‐life of many (NP) databases is short; only few of them are sustainably managed and under continued development. Data quality is always of concern, but when it comes to NPs, extra caution should be exercised, in particular when using the data with computational methods relying on the accurate representation of 3D molecular structures. This is because stereochemical information on NPs is fairly commonly inaccurate or incomplete.

Virtual NP databases can be categorized into (i) encyclopedic and general NP databases, (ii) databases enriched with NPs used in traditional medicines, (iii) specialized databases focused on specific habitats, geographical regions, organisms, biological activities, or even specific NP classes. The largest of all free NP databases is Super Natural II,^[32]^ which consists of more than 325k NPs. The database can be queried via a chemistry‐aware web interface but bulk download is not officially supported. Among the most outstanding free, downloadable resources is the Universal Natural Products Database (UNPD),^[5]^ which lists more than 200k NPs from all forms of life. Unfortunately, this database appears to no longer be hosted. Further large databases include the TCM database@Taiwan,^[33]^ which lists more than 60k NPs found in Chinese medical herbs, the Natural Product Atlas,[^34^, ^35^] offering data on over 25k NPs from bacteria and fungi, and the Collective Molecular Activities of Useful Plants (CMAUP) database,^[36]^ a collection of over 47k NPs from more than 5600 plants with their biological activities information.

**Figure 1:**
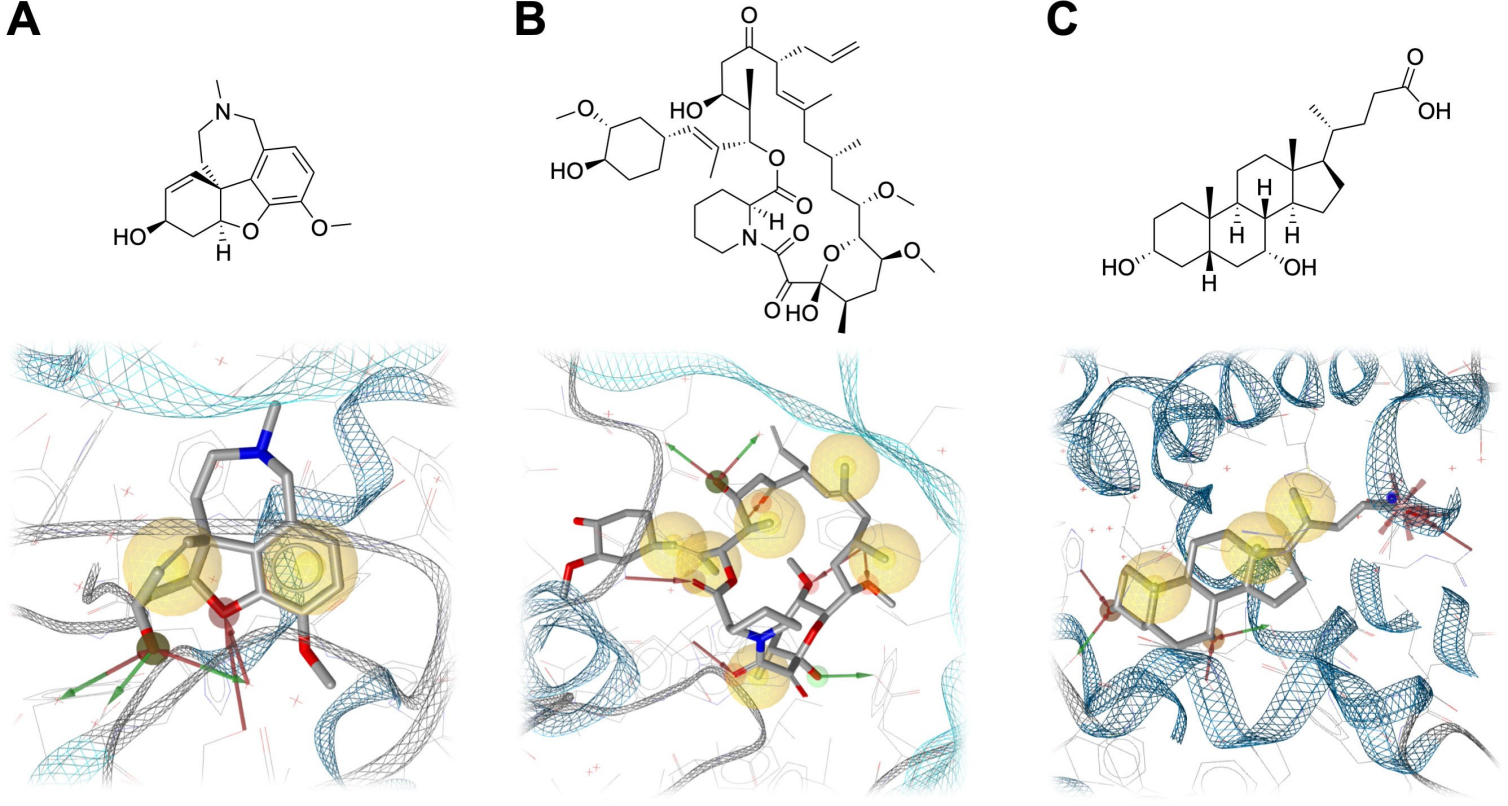
Examples of approved NP drugs and how they bind to their target proteins: (A) (‐)‐galantamine, an acetylcholinesterase inhibitor approved for the treatment of Alzheimer's disease (PDB ID 1DX6), (B) tacrolimus, a macrocyclic immunosuppressant targeting the immunophilin FKBP‐12 (FK506 binding protein; PDB ID 1FKF) and (C) chenodeoxycholic acid, an endogenous bile acid that is used for the treatment of hypocholesterolemia. Chenodeoxycholic acid stimulates the farnesoid X receptor (FXR; PDB ID 6HL1). Carbon atoms grey; oxygen atoms red; nitrogen atoms blue. Hydrogen bonds formed between the ligand and the protein or water molecules are visualized by red arrows (acceptors on the ligand side) and green arrows (donors on the ligand side); hydrophobic features are visualized as yellow spheres, and negative ionizable features as red stars. Visualization and pharmacophore perception with LigandScout.^[39]^

In contrast to information on molecular structures, data on the biological activities and protein‐bound conformations of NPs remain sparse. By overlapping our set of approximately 250k NPs with the full ChEMBL database (a database providing bioactivity data on approximately 2 Million compounds),[^37^, ^38^] we found that only about 16 % were present in the ChEMBL database and had at least one bioactivity annotation.^[31]^ Likewise, by overlapping the NP dataset with all small‐molecule ligands represented in the Protein Data Bank (PDB), we found that for only about 2000 NPs at least one co‐crystallized X‐ray structure of high quality is available.^[6]^ The X‐ray structures of three NPs approved as drugs and bound to their target proteins are shown in Figure 1.

Since the publication of our recent works,[^30^, ^31^] more than one dozen new NP databases have appeared and existing ones have been updated. However, only few of these databases offer bulk download of molecular structures. Among the most relevant databases to mention is the Marine Natural Library,^[40]^ which allows the download of the full dataset of more than 14k marine NPs. In early 2020, a new database was introduced which its authors claim to be the world‘s largest collection of NPs.^[41]^ It should be noted that this database combines data from resources of which some are known to also include substantial numbers of NP derivatives and analogs, and that the data will require additional curation for most applications in cheminformatics.^[41]^

The reader is referred to refs. [30, 31, 41–45] for additional information on NP databases relevant to cheminformatics.

### 2.2 Physical Natural Products Collections

Today, most of the hundreds of compound suppliers worldwide provide comprehensive information on the molecular structures (and other properties) of their compounds for the purpose of virtual screening and other applications free of charge. The majority of the commercial compound collections are dominated by synthetic compounds. By overlapping a comprehensive collection of more than 250k NPs (which we compiled by curating and merging all of the NP datasets available to us^[31]^) with the 7.3 million in‐stock compounds listed in the ZINC database[^46^, ^47^] (a comprehensive database of compounds that are available from various commercial sources and research institutes), we found that only about 10 % of the known NPs (approximately 25k) are readily obtainable for experimental testing.^[31]^ This confirms that the availability of materials for experimental evaluation represents the bottleneck in NP‐based drug discovery. Note that by allowing minor structural deviations between NPs and purchasable compounds, meaning the inclusion of mainly NP derivatives and analogs, the number of readily obtainable compounds increases by roughly 10k to 30k.^[31]^ It is also worthwhile mentioning that the majority of the readily obtainable NPs have physicochemical properties that are considered favorable in the context of drug discovery. In fact, more than half of them are fragment‐sized (molecular weight below 300 Da),^[31]^ hence offering ample opportunities for optimization.

Purified NPs are available from more than 100 commercial providers worldwide^[31]^ but only a dozen of these companies offer more than 5000 NPs. Pure collections of genuine NPs are rare whereas mixed catalogues are commonplace. In these mixed catalogues, however, genuine NPs, NP derivatives and NP analogs are rarely labeled as such. Surprisingly often there is no mention of NPs found on the websites of compound providers, even of those vendors that offer substantial numbers of different NPs. Therefore, tools for identifying NPs and NP‐like compounds can be of high value to NP‐based drug discovery (see Section 6 for details).

The discussion of catalogue sizes should not obscure the importance of compound diversity with respect to physicochemical, structural and biological properties. In this context it is encouraging to know that the (above‐mentioned) 25k readily purchasable NPs cover more than 5700 Murcko scaffolds. We also found that the readily purchasable NPs give a good representation of all of the major NP classes, such as alkaloids, steroids and flavonoids.^[6]^

## 3 Computational Methods for Structure Elucidation and Dereplication of Natural Products

The sourcing of materials for the extraction and isolation of NPs are expensive and time‐consuming, and with increasing knowledge of NPs, the chances for finding novel compounds are diminishing. In order to enable the efficient use of the available experimental resources, analytical and computational methods are utilized in tandem in order to identify known NPs as well as NPs with undesirable properties at the earliest possible point in time.^[44]^ An important component in this interplay of technologies are databases providing measured analytical data (e. g. bioactivities, chromatographic data, mass spectrometry (MS) and nuclear magnetic resonance (NMR) spectroscopy data) for known NPs and their interrogation with computational methods. However, even the largest of these databases cover only a small fraction of the known NPs, for which reason computational methods are increasingly being employed also for the prediction of MS fragmentation and NMR spectra, sometimes in combination with structure generators.^[44]^

There are elaborate algorithms in place which allow the transformation of spectral data into representations (reduced to peak lists, numerical vectors, trees or others) that enable the efficient comparison of spectra and ranking according to their similarity. In other words, these methods have the capacity to identify spectra derived not only from the same compounds but also from structurally related compounds. This means that the applicability of these methods goes beyond known NPs and that they can provide, for example, valuable hints on chemical classes and functional groups. However, such analyses still require manual interaction by an expert, hence limiting automation.^[48]^

A main approach to computer‐assisted dereplication is the combination of analytical data with multivariate data analysis.^[44]^ Using dimensionality reduction techniques such as principal component analysis (PCA), clustering methods, and/or discrimination analysis can help to identify interesting NPs in complex mixtures, e. g. NPs in extracts that are unique to a particular organism of interest.[^49^, ^50^]

Systems for computer‐assisted structure elucidation (CASE) aim to identify the correct structure of a compound of interest based on the available spectroscopic data.^[51]^ More specifically, CASE systems enumerate the structures that are consistent with the experimental (spectroscopic) data and rank them according to their probability. Ideally, CASE systems work in a fully automated fashion, at low error rates. Elaborate CASE systems also take stereospecific NMR data and/or calculations based on density functional theory into account and hence can be used for the assignment of stereochemical properties to NP structures.^[51]^

Machine learning approaches enjoy high interest in NP dereplication. For example, in a recent study the capacity of machine learning algorithms to assign NPs to eight NP classes (such as chromans) based on ^13^C NMR spectroscopy data was explored.^[52]^ The best performance was obtained with an XGBoost classifier. For most NP classes, more than 80 % of the compounds of a test set were correctly assigned. Another study successfully employed a convolutional neural network‐based approach for the rapid identification of new NPs from a filamentous marine cyanobacterium.^[53]^

A different approach is taken by the NP‐StructurePredictor.^[54]^ Based solely on targeted molecular weights derived from *m/z* values obtained by liquid chromatography‐MS, this tool produces a rank‐ordered list of likely NP structures. In order to do so, the tool features a structure generator that can combine the different scaffolds and decorations (which draws from a large NP database), and that can infer structures from structurally related scaffolds.

For more information on experimental and computational methods for NP dereplication readers are referred to recent reviews on this topic, for example, refs. [44, 48, 55, 56].

## 4 Computational Analysis of the Physicochemical and Structural Properties of Natural Products

Cheminformatics has been playing a key role in the characterization of NPs by their physicochemical and structural properties, and in the comparison of NPs with small‐molecule drugs, drug‐like compounds and other types of (organic) molecules. NPs cover a much broader chemical space than synthetic compounds and they populate also areas in chemical space that are generally not (or only with great difficulties) synthetically accessible.[^6^, ^8^, ^19^, ^57^, ^58^] The structural uniqueness (and complexity) of some NPs could allow them to target macromolecules that are otherwise undruggable.^[16]^

NPs are on average heavier and more hydrophobic than synthetic drugs and synthetic, drug‐like compounds.^[59]^ Their structural complexity is also often higher, in particular with regard to stereochemistry (commonly quantified by the number of chiral centers,[^57^, ^59^, ^60^, ^61^, ^62^, ^63^, ^64^, ^65^, ^66^] the number of fraction of Csp^3^ atoms,[^6^, ^8^] and/or the number of bridgehead atoms in ring systems^[67]^) and 3D molecular shape.[^8^, ^68^]

NPs show an enormous diversity of ring systems, in particular of aliphatic systems.[^6^, ^8^, ^57^, ^63^, ^65^] One study showed that 83 % of core ring scaffolds of NPs are absent in commercially available screening databases.^[69]^ With regard to atom composition, two of the most discriminative features of NPs over synthetic compounds are the (on average) low number of nitrogen atoms and high number of oxygen atoms.[^57^, ^59^, ^62^, ^63^, ^64^] Nevertheless, a clear majority of the known NPs, and even more so in physical NP libraries, are drug‐like.^[6]^

NPs from different kingdoms have distinct physicochemical and structural properties.[^66^, ^70^, ^71^, ^72^, ^73^, ^74^, ^75^, ^76^] For example, NPs with macrocycles or long aliphatic chains are more commonly to marine species than terrestrial species.^[74]^ Also bacteria produce many macrocyclic NPs.^[75]^ Their NPs are characterized by a high proportion of heteroatoms and, related to this, a high diversity of functional groups.^[76]^

## 5 Computational Methods for the Assessment of the Structural Diversity of Natural Products

NPs are unrivalled in terms of structural diversity, a fact which is also reflected on a fragment level.^[77]^ Most of the studies assessing the structural diversity of NPs and comparing them to that of synthetic compounds make use of the concept of molecular frameworks (scaffolds) introduced by Bemis and Murcko.^[78]^ In recent work, Ertl and Schuhmann^[75]^ show an intuitive visualization of scaffolds characteristic to NPs and compare them with those of synthetic compounds. They also provide a comparison of scaffolds frequently observed in NPs produced by bacteria, plants, fungi or animals. Rule‐based methods offer a different angle towards NP diversity analysis. They allow, for example, the automated assignment and assessment of the major NP classes.^[6]^

A powerful tool for the intuitive, visual analysis of the structural diversity of sets of compounds is Scaffold Hunter.[^79^, ^80^] The Java‐based, open source software features a graphical user interface and multiple clustering algorithms. Scaffold Hunter is based on the idea of the hierarchical representation and classification of molecular scaffolds (“scaffold tree”). An early version of this tool formed the basis of the structural classification of NPs (SCONP), a method for charting the chemical space of NPs.^[81]^

One of the most commonly employed techniques for mapping the chemical space is PCA,[^6^, ^58^, ^59^, ^64^, ^73^, ^82^, ^83^] which projects high‐dimensional data into a low‐dimensional space for improved interpretability, while keeping information loss to a minimum. The most relevant result of PCA and starting point for interpretation is the PCA scatter plot, which shows the distribution of the data points in the low‐dimensional space. When interpreting a PCA scatter plot it is very important to understand and consider the proportion of variance explained by the shown (two or three) principal components. Only if the proportion of variance explained is sufficiently high, the observed distribution of the data points is informative. This is typically not the case for PCAs based on molecular fingerprints; physicochemical property descriptors usually give better results with PCA.

To avoid the need for the recalculation of the principal components as new compounds are added to the datasets, a method named ChemGPS^[84]^ was developed and extended for use with NPs (“ChemGPS‐NP”^[85]^). The method utilizes predefined rules in combination with selected molecular structures to render a “global drugspace map” into which new structures are projected based on predicted PCA scores. ChemGPS‐NP has been used in several studies for mapping the chemical space of small molecules,[^71^, ^86^] for mode of action prediction,^[87]^ and for the analysis of structure‐activity relationships.[^86^, ^88^]

Also self‐organizing maps and generative topographic maps have been regularly utilized for comparing the molecular structures of NPs with those of drugs, and for visualizing the structural diversity of fragment‐sized and non‐fragment sized NPs.[^66^, ^89^, ^90^] One interesting observation from these analyses is a high degree of resemblance of NPs and synthetic drugs in term of their pharmacophore features, despite profound differences in chemical structure.^[90]^

Further powerful methods for dimensionality reduction include T‐distributed Stochastic Neighbor Embedding (t‐SNE)^[91]^ and the recently introduced Uniform Manifold Approximation and Projection for Dimension Reduction (UMAP) method.^[92]^ t‐SNE produces plots where, overall, similar objects are located in close proximity and dissimilar objects are modeled by distant points. t‐SNE can produce visualizations that are superior to those from PCA but the method does not scale well with the size of data sets. UMAP is conceptually related to t‐SNE and produces similar results but it is faster.

The research group of Medina‐Franco has been developing several methods for the intuitive characterization, visualization and comparison of compound collections, with focus on NP databases. For example, they developed the Consensus Diversity Plot (CDP),^[93]^ which allows the comparison of datasets by a single, straightforward 2D plot representing the median (or other) values of four key properties of choice (e. g. physicochemical property, molecular diversity, scaffold diversity). Each dataset is represented by a single data point. The data point is positioned in the 2D plot according to two properties of choice represented by the x and y axes. The third property of choice is represented by color coding of the data points, and the fourth one (intuitively, this would be the database size) is represented by the size of the data point. The method has been used for the visual comparison of multiple small‐molecule databases[^83^, ^94^, ^95^, ^96^] and is accessible via a web service.^[93]^

Recently, researchers from the same group reported the development of a new method for the representation of the chemical space of compound databases by a single fingerprint called Statistical‐Based Database Fingerprint (SB‐DFP).^[97]^ The SB‐DFP is widely applicable and can be derived, in principle, from any molecular fingerprint and for any reference set. The SB‐DFP is generated by comparing the binomial distributions of features of the molecular fingerprint of choice among the compounds of a dataset of interest and that of a reference dataset. Only bits for which significantly higher “on” rates are observed in the molecular fingerprint among the compounds in the dataset of interest (than in the reference set) will be set to “1” in the SB‐DFP. The SB‐DFP was utilized for assessing and visualizing the similarity of the chemical space of sets of NPs and synthetic compounds, confirming that NP collections cover ample chemical space that remains to be explored (more thoroughly) in the context of drug discovery.

## 6 Computational Methods for the Assessment of Natural Product‐likeness

Computational tools are able to discriminate NPs and NP‐like compounds from synthetic compounds with high accuracy, and they are also able to quantify the NP‐likeness of compounds. As such they are commonly applied to compound design, library design, the selection of NPs (and NP derivatives and analogs) from mixed compound collections, and for compound prioritization.[^59^, ^98^]

**Figure 2:**
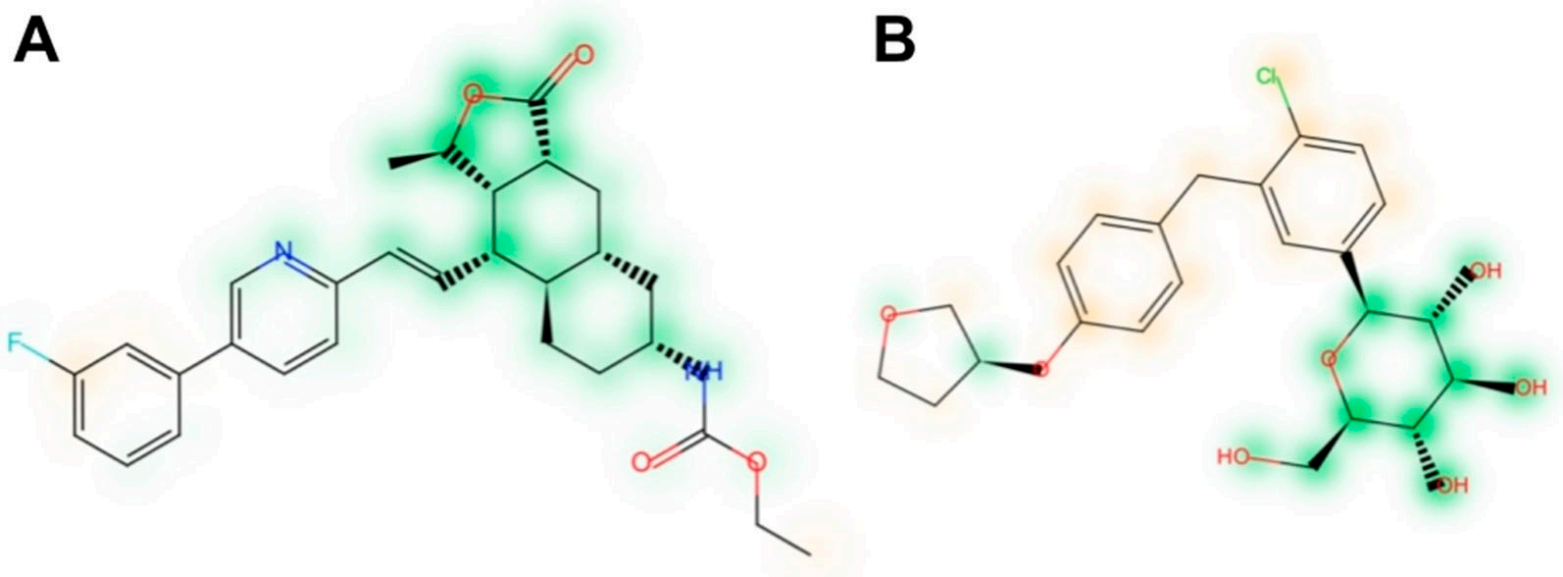
Similarity maps of (A) vorapaxar and (B) empagliflozin. Green‐highlighted atoms contribute to the classification of a molecule as a natural product; orange‐highlighted atoms contribute to the classification of a molecule as a synthetic compound. Adapted from [59] (CC BY 4.0; https://creativecommons.org/licenses/by/4.0).

One of the most established approaches is the NP‐Likeness Score developed by Ertl et al.^[99]^ Employing Bayesian statistics, this score quantifies the NP‐likeness of compounds based on the similarity of their fragments with those of known NPs. The NP‐Likeness Score has been re‐implemented in different software and platforms, with some modifications.[^100^, ^101^, ^102^, ^103^] Further approaches include a conceptually related method employing extended connectivity fingerprints (ECFPs)^[98]^ as well as a rule‐based approach.^[104]^ More recently, we developed NP‐Scout,^[59]^ a tool for identifying NPs and NP‐like compounds in large sets of molecules. The random forest classifiers are trained on a large collection of known NPs and synthetic compounds. On a representative test set, a classifier based on MACCS keys obtained an area under the receiver operating characteristic curve (AUC) of 0.997 and a Matthews correlation coefficient (MCC) of 0.960. NP‐Scout makes use of similarity maps, which highlight areas in a molecule that contribute to the prediction of a molecule as NP or synthetic compound (Figure 2). NP‐Scout is accessible via a free web service.^[105]^

Most recently, the Natural Compound Molecular Fingerprint (NC‐MFP) was introduced as a new approach of describing in particular the structural features of NPs in terms of the scaffolds and fragments they are composed of.^[106]^ The NC‐MFP was shown to outperform established fingerprints in discriminating NPs from synthetic compounds.

## 7 Computational Methods for the Identification of Bioactive Natural Products

Computational methods have a strong track record in the identification of bioactive NPs. The entire range of virtual screening methods has been applied for NP research, from simple, fast methods based on 2D molecular fingerprint similarity to more complex, 3D methods based on molecular shape similarity, pharmacophore models, molecular interaction fields, or docking. More recently, machine learning approaches have become a mainstay in virtual screening for bioactive NPs.^[107]^

In particular 3D virtual screening methods are challenged by the structural properties of many NPs such as high degrees of conformational flexibility, the complexity of their molecular shapes and ring systems (notably macrocycles), insufficiencies of molecular force fields primarily parameterized for synthetic compounds, and uncertainties related to protonation states, tautomerism and oxidation states (for example, the possible involvement of polyphenols in redox cycles is often disregarded). One approach to reduce the structural complexity of NPs is to remove the sugars and sugar‐like components from NPs in cases where they are deemed not to be essential for bioactivity.[^66^, ^108^] This can be done, for example, by use of defined (SMARTS) patterns.[^6^, ^100^]

Given the sparsity of available structural data, docking of NPs to the structures of macromolecules can pose a profound challenge. This is because docking algorithms and scoring functions are highly sensitive even to very small changes in 3D structure such as those commonly induced by ligand binding (including solvent effects). However, also this hurdle may be overcome by the prudent use of homology modeling techniques, induced fit docking approaches, and/or molecular dynamics simulations. In the case of highly flexible proteins, docking against multiple, representative protein structures (“ensemble docking”) may be a good way forward (not only for virtual screening but also for binding mode prediction).[^109^, ^110^] Diligence and patience will certainly be required and, above all, checks of the plausibility of a hypothesis using all available information can help to piece the puzzle together.

More often than in virtual screening‐docking algorithms produce good results in binding mode prediction.^[111]^ Provided that the NP of interest is not excessively large or flexible (as a rough guide, not exceeding 35 heavy atoms or eight rotatable bonds), that the ligand binding site is well‐defined (i. e. not overly shallow, not solvent‐exposed), and that the interaction between the binding partners involves two or more directed interactions, there is a good chance that a sufficiently accurate binding pose can be obtained that offers crucial insights for the development of optimization strategies. Binding pose prediction is more feasible than virtual screening because it allows to largely disregard the most challenging aspect of docking, which is the scoring of compounds according to their binding affinity, and it allows researchers to focus their effort on one specific ligand‐target pair. Importantly, in particular in the context of NP research, docking enables the rationalization of stereoselectivity in ligand binding (and other processes, such as metabolism). The importance of using the correct stereochemical information with 3D approaches, especially with docking, cannot be overstated.

In the following paragraphs we briefly discuss representative examples of studies in which virtual screening was successfully employed for the identification of bioactive NPs. For more comprehensive discussion of applications, the reader is referred to excellent reviews.[^18^, ^112^]

**Figure 3:**
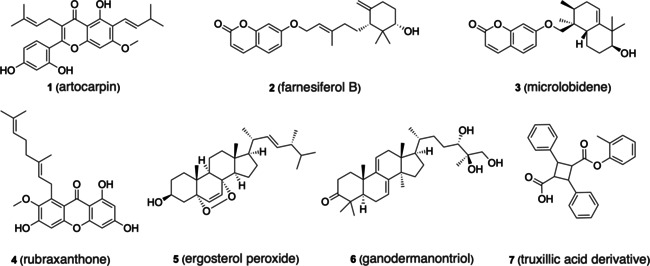
Examples of natural products and natural product derivatives identified by virtual screening.

Using katsumadain A (a diarylheptanoid inhibiting influenza neuraminidase) as a template for 3D molecular shape‐based screening, a number of structurally distinct NPs were identified that inhibit the viral enzyme with IC_50_ values in the submicromolar to low micromolar range (for example artocarpin (**1**), which is depicted in Figure 3).^[113]^ In another study, pharmacophore‐based virtual screening was combined with a shape‐based approach in order to identify activators of the G protein‐coupled bile acid receptor 1 (GPBAR1).^[114]^ In addition to several NP databases also a collection of synthetic compounds was screened. Among the 14 selected NPs eight (57 %) obtained a measured receptor activation of at least 15 % at 20 μM concentration. Two of these compounds, farnesiferol B (**2**) and microlobidene (**3**), are based on molecular scaffolds that had not yet been associated with GPBAR1 modulation. Both compounds were reported to have EC_50_ values of approximately 14 μM. Among the 19 selected synthetic compounds, only two were active (applying the identical activity threshold).

Influenza neuraminidase has also been successfully addressed by docking. For example, a database of NPs related to plants endogenous to Malaysia was screened for potential inhibitors of influenza neuraminidase.^[20]^ From the five plants with the highest hit rates in docking, twelve NPs with moderate inhibitory activity on influenza neuraminidase were identified by experimental testing (one example is rubraxanthone (**4**)), four of which had been ranked by docking among the top‐100 compounds in the hit list.

A pharmacophore approach was utilized to screen a collection of 10k NPs related to traditional Chinese medicine for compounds targeting the farnesoid X receptor (FXR), a transcription factor involved in inflammatory liver diseases.^[115]^ Screening results indicated a high likelihood of activity of lanostane triterpenes from the mushroom *Ganoderma lucidum*. Several of these lanostanes were isolated and subjected to experimental testing in a reporter gene assay. Five lanostanes showed a dose‐dependent induction of FXR with EC_50_ values in the low micromolar range, the most active ones being ergosterol peroxide (**5**) and ganodermanontriol (**6**).^[21]^

Rupp et al.^[116]^ explored a number of different machine learning approaches in order to identify NP derivatives that selectively activate the peroxisome proliferator‐activated receptors (PPARγ). The authors focused on the use of Gaussian process models (with different kernels) that they employed to learn pharmacophoric patterns from a medium‐sized set of synthetic PPARγ ligands. By screening and ranking several hundred thousand commercially available compounds, the authors identified a truxillic acid derivative (**7**) as a selective activator of PPARγ (EC_50_=10 μm).

Another study from the same lab^[117]^ employed machine learning‐based virtual screening for the identification of mimetics of the Alzheimer drug (−)‐galantamine (Figure 1). Like for many Alzheimer drugs, the therapeutic efficacy of (−)‐galantamine is linked to activities on multiple proteins rather than a single one. In the search for efficacious compounds it is hence important to consider polypharmacology. To this end, Grisoni et al. employed the machine learning‐based target prediction models SPIDER and TIGER (which are discussed in more detail in the next section) to identify (in this case synthetic) compounds with bioactivity spectra that are comparable to that of (−)‐galantamine. Using these models, they selected 20 compounds from a set of more than 3 Million purchasable compounds for testing. Among the selected compounds, several showed interesting activities in vitro. Two compounds of small size were shown to have polypharmacological profiles that are considered to be favorable for the treatment of Alzheimer's disease.

## 8 Computational Methods for the Prediction of the Macromolecular Targets of Natural Products

Knowing the macromolecular target(s) of small molecules is of utmost importance to the assessment of the pharmacological efficacy and safety of compounds, and for their further development. However, even for a substantial number of marketed drugs the mode of action is unknown or only vaguely understood. The road to the experimental identification of the target(s) of small molecules can be very lengthy and expensive, and there is a good chance to be met by disappointment on the way, for example, when it becomes clear that “the target” of a supposedly innovative compound is an established drug target or, worse, a protein known to be not a viable drug target. Computational approaches are hoped to make a significant contribution to making mode of action identification more efficient and there is an increasing body of evidence that some of these hopes are becoming reality (as will be discussed below).

In silico target prediction can be regarded as a large‐scale application of virtual screening (see the previously discussed study of Grisoni et al.^[117]^), in the way that one, several or many compounds are screened against the widest possible set of macromolecules. A plethora of methods and models have been reported in recent years[^118^, ^119^, ^120^, ^121^] and they have become established as important tools in early drug discovery. Related to the challenges involved in docking and structure‐based methods in general (in particular, the limited coverage of macromolecules by the available structural data), most approaches for target prediction are ligand‐based.

Ligand‐based methods cover the full range from straightforward similarity‐based approaches to complex machine learning and network‐based approaches. Surprisingly, despite today‘s abundance of computational methods for target prediction, our understanding of the value of these methods under real‐world conditions remains limited.^[122]^ This is primarily because of the (in general) prohibitive costs involved in the experimental, systematic, prospective evaluation of such models, but also because of the partly insufficient, superficial retrospective validation protocols that are regularly employed.[^122^, ^123^] To our best knowledge, the only computational method for which a systematic experimental validation has been reported so far remains the well‐known Similarity Ensemble Approach (SEA).[^124^, ^125^, ^126^] One may rightly argue that validating models on existing data generally leads to an overestimation of how well a model will perform under real‐world conditions, however, there is at least one more important point to consider when judging the value of target prediction approaches based on retrospective validation studies: under real‐world conditions, researchers will rarely face the situation where no hints on a compound‘s target are available at all. A scenario where a substantial amount of information is available on a compound of interest, e. g. phenotypic assay readouts with different cell lines or data for structurally related compounds, is more likely. By adding up all of the available information it is likely that many false‐positive predictions can be ruled out, hence leaving much fewer candidate targets to be investigated experimentally.

In a recent, in‐depth study of the performance and scope of a similarity‐based approach and a machine learning approach for predicting the targets of small molecules, we show that the reliability of predictions of either approach strongly depends on the structural relationship between the compounds of interest and compounds represented in the training set (or knowledge base).^[123]^ This fact needs to be carefully considered when working with NPs, given the fact that models for target prediction are mostly designed for, and trained on, measured data for synthetic compounds.

In the same study we found that, surprisingly, with the currently available data, the similarity‐based approach generally outperformed the machine learning approach. While a direct comparison of these two approaches should, for several reasons, be considered with great caution, the results suggest that the simple similarity‐based approach is a good choice, in particular also when taking into account model interpretability. This is also reflected by the good performance of other established, similarity‐based models such as SwissTargetPrediction.^[127]^

Most NPs are structurally distinct from more conventional, synthetic compounds, which account for the bulk of the measured activity data. More complex similarity‐based methods that compare molecules based on their 3D molecular shape are designed to recognize such distant structural similarity but until recently it was unclear how well these methods would work in practice. We systematically explored the capacity of ROCS,[^128^, ^129^] a leading, shape‐based screening engine that also takes into account chemical feature distributions, to identify the macromolecular targets of “complex” small molecules based on a knowledge base of “non‐complex” compounds with measured bioactivity data.^[130]^ For the purpose of this work, we defined molecules as “complex” if they are either (very) large in size (45 to 55 heavy atoms) or macrocyclic (and large). In contrast, we defined molecules as “non‐complex” if they were small in size (15 to 30 heavy atoms). A total of 28 pharmaceutically relevant targets were studied. For each of the targets a diverse set of 10 complex small molecules was automatically generated. A single, low‐energy conformation of each of these molecules was used as a query for screening with ROCS against a multi‐conformational knowledge base. The knowledge base represents 3642 targets with a total of 272 640 non‐complex small molecules. This study found that ROCS correctly ranked at least one known target among the top 10 positions (out of a list of 3642) for up to 37 % of the 280 complex small molecules serving as queries. Considering the dissimilarity of the queries and the compounds in the knowledge base, this performance is remarkable. It indicates that target prediction is possible for a substantial number of challenging complex molecules. Note that researchers will be able, in many cases, to strongly reduce the number of target candidates based on expert knowledge and available information. Among the 280 complex small molecules were at least 31 known, complex NPs and NP‐like compounds. For these compounds, the top‐10 success rate was lower (23 % vs. 37 %). This is related to the fact that the median Tanimoto coefficient based on Morgan2 fingerprints of the complex NP (or NP‐like compound) and the closest non‐complex small molecule in the knowledge base is only 0.13. For pairs of compounds sharing such a low degree of similarity it can be expected that their binding modes are distinct, which is generally beyond the scope of ligand‐based methods. In summary, taking into account capacity of these methods and their low demand in computational power, we believe it is worthwhile using these methods in any case as valuable ideas may emerge from their use.

Besides 3D similarity‐based approaches, also 3D pharmacophore‐based approaches are regularly used for target prediction in the context of NP research. One example is a profiling study in which secondary metabolites isolated from the medical plant *Ruta graveolens* were screened against a battery of more than 2000 pharmacophore models representing over 280 targets.^[131]^ From this in silico screen, among other bioactive NPs and interactions, arborinine was identified as an inhibitor of acetylcholinesterase (measured IC_50_=35 μM).

In recent years the models for NP target prediction which have seen most interest certainly are those based on machine learning. Notable examples include SPiDER,^[132]^ TIGER,^[133]^ and STarFish.^[134]^ SPiDER uses self‐organizing maps in combination with “fuzzy” molecular descriptors that allow for extending its usage to NPs.[^135^, ^136^] The model was instrumental in the identification of 5‐lipoxygenase, PPARγ, glucocorticoid receptor, prostaglandin E2 synthase 1, and FXR as targets of the macrolide archazolid A,^[137]^ and it correctly predicted prostanoid receptor 3 as a target of doliculide, a 16‐membered depsipeptide.^[138]^ SPIDER also successfully identified the targets of several fragment‐like NPs such as (i) sparteine, for which the kappa opioid receptor, p38α mitogen‐activated protein kinase, muscarinic and nicotinic receptors were experimentally confirmed as targets,^[3]^ (ii) DL‐goitrin, for which the pregnane X receptor and the muscarinic M1 receptor were experimentally confirmed as targets,^[139]^ (iii) isomacroin, for which the platelet‐derived growth factor receptor and the adenosine A_3_ receptor were experimentally confirmed as targets,^[139]^ and (iv) graveolinine, for which cyclooxygenase‐2 and the serotonin 5‐HT_2B_ receptor were experimentally confirmed as targets.^[139]^

Building on predictions from SPiDER, the Drug‐Target Relationship Predictor (DEcRyPT)^[140]^ employs random forest regression in order to generate a refined list of likely macromolecular targets. Use of DEcRyPT led to the successful identification of 5‐lipoxygenase as a target of the ortho‐naphthoquinone β‐lapachone.^[140]^ The hydroquinone form of β‐lapachone was confirmed as a nanomolar inhibitor of 5‐lipoxygenase.

TIGER is conceptually related to SPiDER. However, it employs modified CATS descriptors and uses a different method for scoring the predicted targets (taking into account ensemble similarity). TIGER successfully identified the orexin receptor, glucocorticoid receptor, and cholecystokinin receptor as targets of the marine NP (±)‐marinopyrrole A.^[133]^ The model also rightly predicted, among other proteins, estrogen receptors α and β as targets of the stilbenoid resveratrol.^[141]^

STarFish is a stacked ensemble approach for target prediction trained on synthetic compounds. Various machine learning algorithms were explored as part of the development process. The best stacking approach identified by the authors used molecular fingerprints as input for a random forest model and a k‐nearest neighbors model (level 0). The probabilities predicted by these two models for each of the targets are then used as input for a meta‐classifier based on logistic regression (level 1). The stacking approach was found to perform substantially better on a test set of NPs (ROC AUC 0.94; BEDROC score 0.73) than the individual models (AUCs between 0.70 to 0.85; BEDROC scores between 0.43 and 0.59).^[134]^

Also network approaches focused on the prediction of the macromolecular targets of NPs have been reported. For example, Cheng and co‐workers developed statistical network models in order to link NPs to anti‐cancer targets^[142]^ and proteins involved in aging‐associated disorders.^[143]^

Most recently, multi‐task deep neural networks were trained on medical indication data and employed for identifying privileged molecular scaffolds in NPs (in this case, scaffolds for which multiple NPs built on the identical scaffold are active in the same indication).^[144]^ Based on the predictions of these models, a privileged scaffold dataset for 100 indications was compiled that could serve as a starting point for NP‐based drug discovery.

For additional information on this topic, the reader is referred to refs. [18,19,145].

## 9 Computational Identification of Natural Products Likely to Interfere with Biological Assays

The inclination of NPs to cause interference with biological assays continues to pose a significant challenge to the experimental screening of NPs.[^146^, ^147^] The flavonoid quercetin, a known aggregator and pan‐assay interference compound, gives an illustrative example of the scale of the problem: as of July 28, 2020, the PubChem Bioassay database listed quercetin as conclusively active in more than 800 unique bioassays, which represents a hit rate of more than 50 % (among all conclusive assay outcomes).

By far the most commonly observed mechanism of assay interference is aggregate formation, which occurs under specific assay conditions.^[148]^ Further relevant mechanisms are covalent binding, redox‐cycling, membrane disruption, metal chelation, interference with assay spectroscopy, and decomposition in buffers.^[149]^

The development of computational approaches aiming to tackle this problem has been slow. Until recently, tools accessible to users included several rule sets, few similarity‐based approaches, and a statistical approach. Among the rule sets, the best known and most applied collection is the pan‐assay interference compounds (PAINS) rule set.[^149^, ^150^] Although clearly declared by its inventors, users of the PAINS rules set all too often neglect the significant limitations of its scope, applicability and reliability. Further examples of relevant rule sets include the REOS rules^[151]^ and a set of rules derived from an NMR‐based method for identifying small molecules that cause false‐positive assay outcomes due to reactivity (ALARM NMR).^[152]^

A useful similarity‐based approach is Aggregator Advisor, which flags compounds which are in a close structural relationship to known aggregators (a simple approach of which negative outcomes of course do not indicate the benignity of compounds).^[153]^ The statistical approach, called BADAPPLE,^[154]^ calculates a promiscuity score based on molecular scaffolds.

More recently, we introduced Hit Dexter 2.0, the second generation of a set of machine learning models that are designed to identify compounds that are likely to show frequent hitter behavior in primary screening assays and/or confirmatory dose‐response assays, regardless of the underlying (interference) mechanism.^[155]^

All these approaches have in common that they are derived from datasets dominated by synthetic compounds. As we point out in our work on Hit Dexter 2.0, the training set, even though consisting of about 250k compounds, covers only a small fraction (approximately 15 %) of the known NPs with compounds that are structurally sufficiently similar so that reliable predictions by the model can be expected.^[155]^ This means, once again, that caution must be exercised when using any of these approaches in particular in the context of NPs.

## 10 De Novo Design of Nature‐inspired Compounds and Compound Collections

Limited synthetic accessibility poses a major challenge to the exploration and use of NPs and NP‐derived compounds.[^19^, ^156^] In order to overcome this hurdle, researchers have devised a number of strategies for the design of synthetically accessible compounds with NP‐like properties. For example, diversity‐oriented synthesis (DOS) is a concept that utilizes pairs of complexity‐generating reactions to produce diverse and complex compounds with NP‐like architectures (enriched with stereogenic centers and sp^3^‐hybridized atoms).[^156^, ^157^] In contrast to DOS, biology‐oriented synthesis (BIOS) starts from biologically active scaffolds and seeks to generate small to medium‐sized collections of complexity‐reduced, NP‐like compounds.[^80^, ^158^] BIOS is guided by the hierarchical representation and classification of molecular scaffolds, as well as the structural similarity of the ligand‐sensing cores of proteins.[^81^, ^159^]

A further strategy for the efficient synthesis of diverse, NP‐like compounds utilizes chemoselective reactions for the distortion of ring systems that are part of readily available NPs.[^160^, ^161^] Common conversions in this context include ring cleavage, ring expansion, ring fusion and ring rearrangements.

Novel classes of compounds can also be derived by fragment‐based compound design starting from NP‐derived fragments.^[156]^ This NP‐inspired strategy may enable the efficient exploration of the biologically relevant chemical space beyond the known NPs and NP scaffolds.

Shifting the focus to computational approaches, Hartenfeller et al.^[162]^ developed DOGS, a de novo design tool which utilizes information on more than 25k readily available synthetic building blocks in combination with a large set of established reaction rules to generate compounds which are likely synthetically accessible. Importantly, DOGS utilizes structural and pharmacophoric descriptions of (bioactive) reference compounds in order to steer the compound generation process into desired directions.

**Figure 4:**
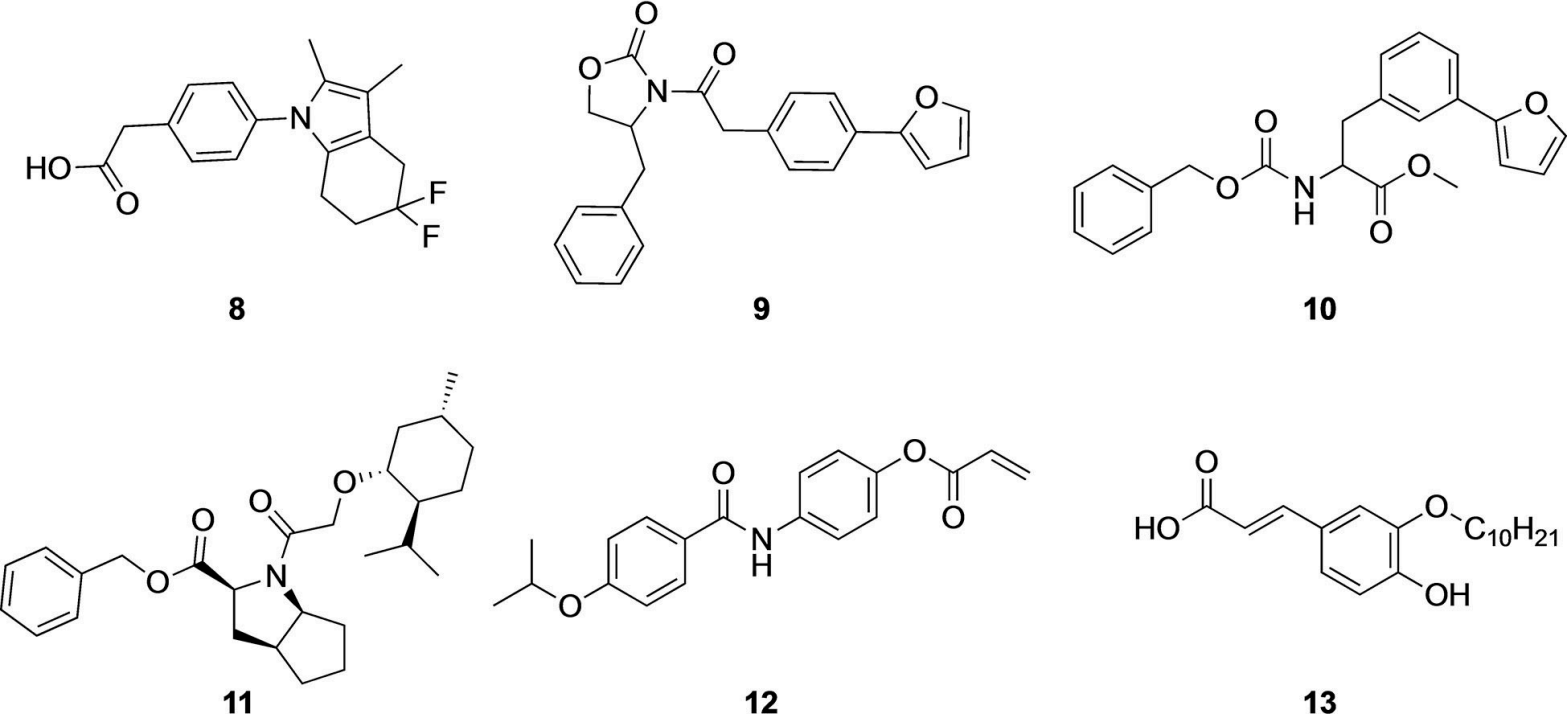
Examples of de novo designed molecules inspired by natural products.

Starting from NPs active on the retinoid X receptor (RXR), DOGS was employed for the design of novel, synthetically accessible, NP‐inspired RXR ligands. Five out of six compounds designed by DOGS proved to be RXR agonists and to have similar nuclear receptor selectivity profiles to the respective templates (one example is **8**, shown in Figure 4).^[135]^ In a further study, DOGS was utilized for the design of mimics of (−)‐englerin, a complex sesquiterpene with potent anti‐proliferative activity.^[163]^ A total of 323 unique designs were generated by DOGS. After several filtering and scoring steps, two proposed molecules (**9** and **10**) were selected and synthesized (one thereof with a slight modification). Both compounds were confirmed in a functional, cell‐based assay as potent inhibitors of the transient receptor potential melastatin 8 (TRPM8) ion channel.^[164]^

In a follow‐up study, the above‐mentioned ranking approach was extended to take into account also the 3D molecular shape similarity (based on global fractal dimensionality) of the 323 designs.^[165]^ One of two compounds selected by this approach (**11**) was again confirmed as potent inhibitor of TRPC4 and TRPM8 channels.

Merk et al. used a deep recurrent neural network approach for the de novo design of RXR modulators.^[166]^ The neural network was trained on synthetic compounds with measured bioactivities on RXR. By fine‐tuning the model with a small set of NPs modulators of RXR, the authors showed that their model was able to produce synthetically accessible NP mimetics that have a high chance of being active on the intended target. Following a selection procedure that involved target prediction and the assessment of molecular similarity, three designs were selected for experimental testing of which two compounds (**12** and **13**) were confirmed to modulate the RXR with a potency that is comparable with that of the templates.

For additional information on de novo design in the context of NP research, the reader is referred to ref. [19].

## 11 Computational Prediction of ADME and Safety Profiles of Natural Products

NP‐based drug discovery often faces challenges related to the ADME and safety profiles of NPs. Among the most prominent examples of anti‐targets addressed by NPs is the hERG channel^[167]^ (its blockage is linked to potentially fatal cardiac arrhythmia), cytochrome P450 enzymes (which can cause drug‐drug interactions and toxicity), and the P‐glycoprotein (an efflux pump with broad substrate specificity that can effectively cause drug resistance). A plethora of computational models of different kinds (i. e. statistical models, machine learning models, pharmacophore models, docking, etc.) address these and many other anti‐targets and endpoints.[^96^, ^168^, ^169^, ^170^, ^171^, ^172^, ^173^] However, it is important to consider that, as a result of the available data, these and most other in silico models are trained and/or tested on compounds that are primarily of synthetic origin. Therefore, extra caution must be exercised in relation with NPs, and the applicability domain of the models must be closely observed.

Not all models are equally affected by the structural and physicochemical differences of NPs and synthetic compounds. For example, the applicability of Hit Dexter 2.0 to NPs is limited. The reliability of Hit Dexter's predictions has been shown to decrease substantially when moving away from the training data beyond a certain point, and the training data are primarily composed of synthetic compounds. In contrast, a conceptually related machine learning model for the prediction of the sites of metabolism of small molecules, FAME 3, was shown to perform well on NPs, even though the majority of compounds in the training set are again of synthetic origin.^[174]^ The reason for the high robustness of the FAME 3 models and their good performance on NPs is that the liability of atom positions in molecules is described based on their proximate atom environment, and these proximate neighborhoods are much more redundant among NPs and synthetic compounds than their global molecular similarity.

## 12 Summary

NPs pose some extraordinary challenges to experimentalists and theoreticians alike, but statistics on recently approved, small‐molecule medicines show that the research of NPs is worth all the effort and can yield valuable, innovative drugs. Modern in silico methods can make a substantial contribution to the acceleration and de‐risking of NP‐based drug discovery. However, the applicability of models must be closely observed, in particular when working with NPs as computational approaches are mostly designed for, and trained on, data for synthetic compounds. Unfortunately, even the recently developed models still often lack robust definitions of the applicability domain and do not warn users adequately about compounds for which predictions are not reliable. Researchers may in particular feel tempted to use one of the many free, user‐friendly web servers to quickly predict physicochemical or biological properties of NPs. Obviously, also for these web services the principle holds true that in the absence of robust indicators of the reliability of individual predictions, these predictions are not to be trusted.

Given the reinvigorate interest in NP research, the growing amount of accessible biological, chemical and structural data, and advances in algorithms, modeling techniques and computational power, the future will see the continued integration of computational methods in NP‐based drug discovery pipelines.

## Conflict of Interest

None declared.

## Biographical Information

*Ya Chen is a Ph.D. student with Ass.‐Prof. Johannes Kirchmair at the Center for Bioinformatics (ZBH) of the Universität Hamburg. She received her bachelor's degree in pharmacy from Jilin University (2013) and her master's degree in medicinal chemistry from Peking University (2016). Her research is focused on the development and application of computational methods for the identification of bioactive natural products and the prediction of their biomacromolecular targets*.

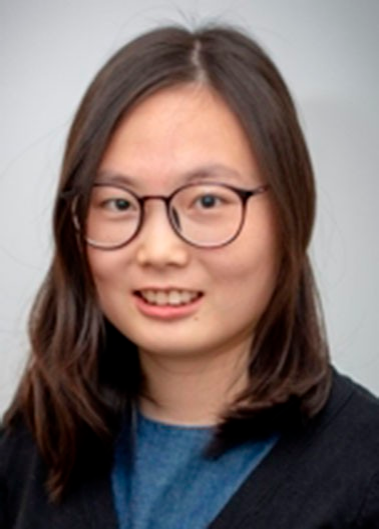

## Biographical Information

*Johannes Kirchmair is an assistant professor in cheminformatics at the Department of Pharmaceutical Chemistry of the University of Vienna and head of the Computational Drug Discovery and Design Group (COMP3D). He also is a group leader at the Center for Bioinformatics (ZBH) of the Universität Hamburg. After earning his PhD from the University of Innsbruck (2007), Johannes worked in different capacities at Inte:Ligand GmbH (Vienna), BASF SE (Ludwigshafen), the University of Cambridge and ETH Zurich. He also held a junior professorship in applied bioinformatics at the Universität Hamburg (2014 to 2018) and an associate professorship in bioinformatics at the University of Bergen (2018 to 2019)*.